# Temperature and Rainfall Shape the Breeding Ecology of Barn Swallows *Hirundo rustica* in an Arid Region

**DOI:** 10.1002/ece3.72661

**Published:** 2025-12-19

**Authors:** Fatima Gherbaoui, Taqiyeddine Bensouilah, Emilio Pagani‐Núñez, Moussa Houhamdi

**Affiliations:** ^1^ Faculty of Nature and Life Sciences, Laboratory Health and Environment University of Bordj Bou Arreridj El Anceur Algeria; ^2^ Faculty of Nature and Life Sciences, Ecology Department University of Bordj Bou Arreridj El Anceur Algeria; ^3^ Laboratory Biology, Water and Environment University of Guelma Guelma Algeria; ^4^ Centre for Conservation and Restoration Science Edinburgh Napier University Edinburgh UK; ^5^ School of Applied Sciences Edinburgh Napier University Edinburgh UK; ^6^ Faculty of Nature and Life Sciences, Department of Nature and Life Sciences University of Guelma Guelma Algeria

**Keywords:** climate, *Hirundo rustica*, rainfall, renesting probability, reproduction success, temperature

## Abstract

Local weather conditions play a critical role in shaping avian reproduction, yet our understanding of these patterns in arid environments, where climate change is expected to increase drought frequency, is limited. This study investigated the effects of temperature, rainfall, and wind speed on the breeding ecology of Barn Swallows ( *Hirundo rustica* ) in an arid region of Algeria during 2023 and 2024. Using generalized linear mixed models (GLMMs), we assessed these effects for several life‐history traits and breeding parameters including clutch size, incubation duration, hatching success, nestling period duration, and reproductive success. Our results indicate that clutch size decreased seasonally, while incubation duration increased with rainfall and was shorter for second clutches. Hatching success correlated positively with the amount of precipitation and was higher in second clutches. Nestling period duration increased both seasonally and with prolonged incubation. Reproductive success benefitted from greater rainfall but declined as the season progressed and when incubation was extended. Additionally, higher temperatures reduced renesting probability, suggesting that heat may limit Barn Swallows' reproductive efforts. Our results highlight the importance of rainfall in shaping reproductive success and reveal the negative impact of high temperatures on the breeding performance of Barn Swallows in this region. In a context of climate change and increasing drought frequency, these findings underscore the challenges that Barn Swallow populations might face in arid environments in the near future.

## Graphical abstract

Here, we show that the breeding ecology of Barn Swallows *Hirundo rustica* in an arid region in northern Algeria is strongly influenced by temperature and precipitation. These effects were apparent in terms of the duration of the breeding stage and on breeding fitness.

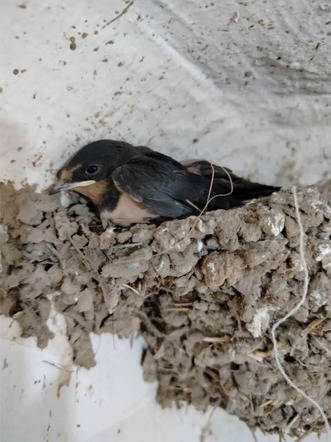

## 1 Introduction

Living organisms, particularly short‐lived species such as birds, are strongly influenced by environmental conditions, with local weather playing a critical role in regulating avian breeding ecology (Both et al. 2006). Weather parameters, particularly temperature and precipitation, affect multiple aspects of reproduction including clutch size, incubation duration, nestling growth, and reproductive success (Coe et al. 2015; Cavalcanti et al. 2016; Gillette et al. 2021). While these effects are highly variable in different climatic regions, high temperatures and increased drought seem to have particularly important effects across species and latitudes (La Sorte et al. 2014; Cohen et al. 2020). These potential negative effects might be exacerbated for birds breeding in arid environments. These negative effects are particularly severe in arid lands, where birds face extreme temperatures, highly variable precipitation, scarcer water and food resources, requiring both behavioral and physiological adaptations to maximize reproductive success under unpredictable and harsh conditions (Cavalcanti et al. 2016; França et al. 2020; Murphy et al. 2022).

Local weather, particularly temperature and rainfall, and food availability strongly influence reproductive success and nesting strategies of bird species, especially in arid zones, where environmental conditions are more restrictive (Bensouilah and Barrientos 2021; Oñate et al. 2023). On the one hand, studies have shown that higher temperatures can accelerate embryonic metabolism, leading to shorter incubation periods (DuRant et al. 2013). On the other hand, excessive heat, especially in arid zones, can also cause thermal stress, resulting in increased embryo and nestling mortality (Bourne et al. 2020). Precipitation is a key factor influencing bird reproduction, especially in arid environments. It plays a crucial role in determining the availability of insects, which are the main food source for Barn Swallows (Dawson et al. 2005). Increased precipitation can lead to enhanced food availability, allowing parents to feed their young more effectively and improve their physical condition (Cavalcanti et al. 2016; França et al. 2020).

Conversely, a lack of rainfall early in the season can limit food resources and force birds to reduce their reproductive effort (Nooker et al. 2005). Wind speed affects insect availability and the foraging efficiency of swallows and other insectivorous birds primarily because wind influences insect flight behavior and abundance, which in turn impacts prey accessibility for these birds (Møller 2013). In multi‐clutch species, a shorter incubation period and a longer nestling phase later in the season may reflect a trade‐off between the need to complete the breeding cycle before migration and optimizing offspring survival (Verhulst and Nilsson 2008). However, despite their high ecological plasticity, there is a significant knowledge gap about how birds' reproductive performance responds to local weather variation in arid zones, especially in northern Africa. This is especially important given the expected negative effects of climate change on desert avian communities (Ma et al. 2023).

The Barn Swallow ( *Hirundo rustica* ) is an excellent biological model for studying the effects of local weather on reproduction. This migratory species breeds in a wide range of environments (e.g., Zhao et al. 2022), including arid regions where environmental conditions can be especially challenging. Its seasonal reproductive cycle, often involving multiple successive clutches, can help us understand how birds adjust their reproductive behavior in response to seasonal climatic variations (Cavalcanti et al. 2016; Teglhøj 2017; Gillette et al. 2021). Thus, this study aimed to understand how extreme aridity affects breeding birds by examining how temperature, precipitation, and wind speed shape key reproductive traits of the Barn Swallow in an arid environment in Algeria. More specifically, we aimed to answer the following questions:

1. How do local weather conditions (temperature, precipitation, wind) and breeding phenology influence the duration of incubation and nestling stages of Barn Swallows in this arid environment?
2. What are the consequences of variation in breeding stage duration and local weather conditions on hatching success, renesting probability, and overall breeding success?

## 2 Materials and Methods

### 2.1 Study Area

**FIGURE 1:**
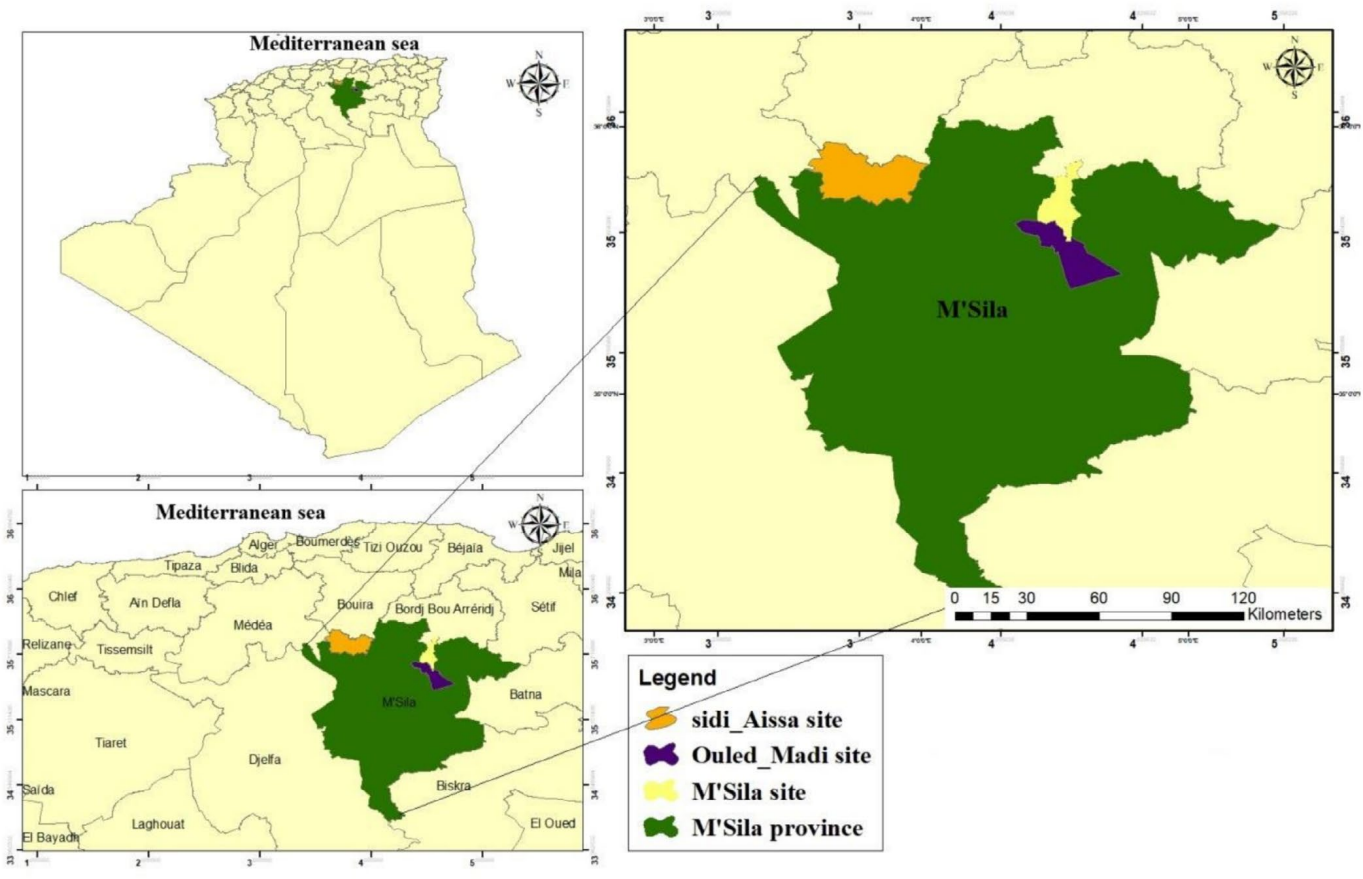
Map of the study area in northern Algeria, highlighting key sites (Sidi Assa, Ouled Madi, and M'Sila) within M'Sila Province. Geographic features such as the Mediterranean Sea and neighboring provinces are labeled for context. The legend highlights study sites within M'Sila Province.

**FIGURE 2:**
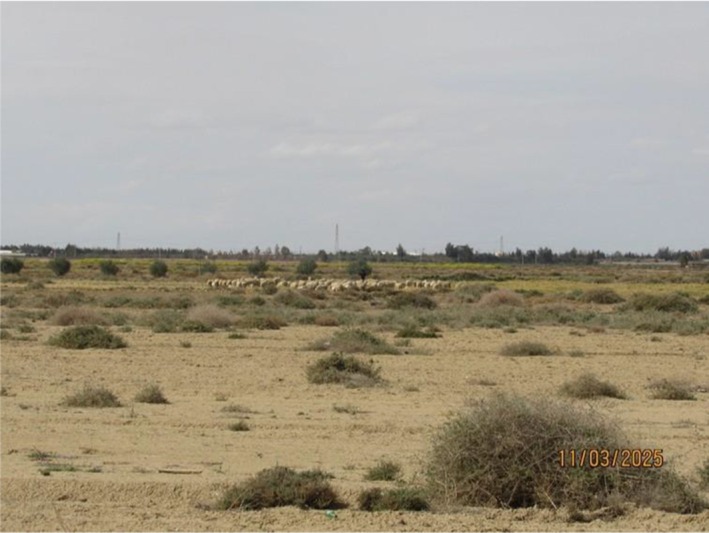
Photograph of the arid plateau landscape in one of the study areas in M'Sila Province, Algeria. The image highlights the region's characteristic topography and sparse vegetation, typical of the semi‐arid to arid conditions in the region.

**FIGURE A1:**
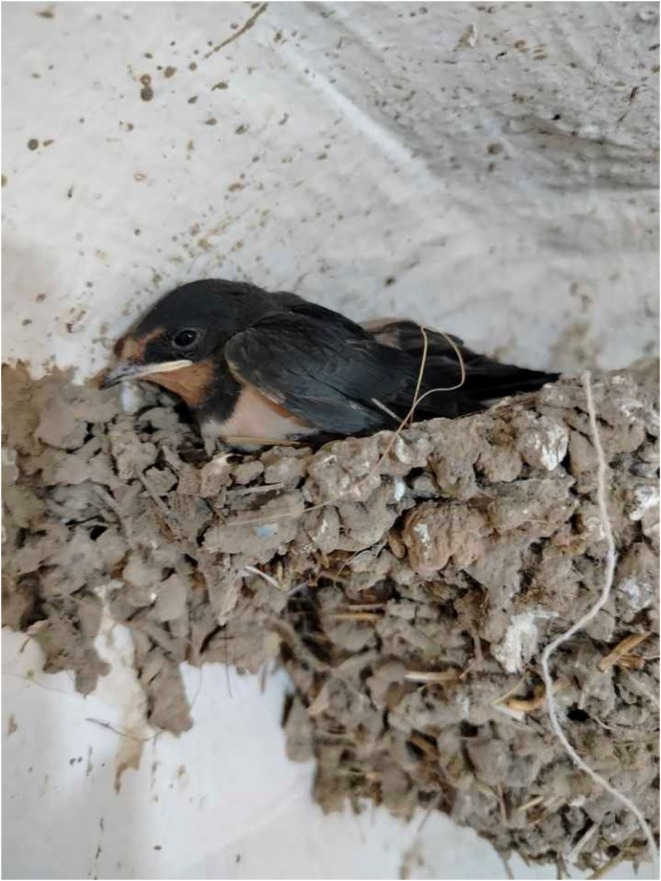
Typical Barn Swallow *Hirundo rustica* nest with nestlings in our study area.

This study was conducted in the M'sila Province from early March to the end of August during two consecutive breeding seasons (2023, 2024). We performed systematic surveys to identify all accessible nests across three administrative communes: M'sila, Ouled Madi, and Sidi Aissa (Figures 1, 2). Nests were found in small villages mostly surrounded by crops, pastoral lands, and arid steppes (Figure A1). We identified 14 subsites with several breeding pairs (M'sila, *n* = 7; Ouled Madi, *n* = 6; and Sidi Aissa, *n* = 1). Main crops were cereals, mostly irrigated. The wilaya (i.e., Province) of M'sila (35°70′58″ N, 4°54′19″ E), located in the heart of the steppe highlands (high‐elevation plateau) of central Algeria at 471 m.a.s.l., is characterized by a semi‐arid to arid climate, marked by high interannual variability in rainfall and an increasing frequency of drought episodes. Its climate features cool to cold winters with mean minimum 4.26 and maximum 14.7°C temperatures, and dry summers when the temperature often exceeds 40°C. Rainfall is low and irregular, typically ranging from 150 to 250 mm per year, which has a significant impact on the region's biodiversity. These harsh climatic conditions have a significant impact on local hydrological dynamics and agropastoral practices.

In the wilaya of M'sila, there is a wide range of land uses: arboriculture (apricots, olives, and apples), forage crops, and intensive vegetable farming in irrigated zones, with rain‐fed extensive farming in peripheral areas (Tir 2024). The hydrographic network, though relatively dense, is predominantly composed of temporary rivers (oueds) depending directly on scarce, irregular and often torrential rainfall. As a result, the main watercourses, which all drain toward the central depression of the region, are mostly dry throughout the year. The wilaya's population reached 1.36 million in 2020, with an average density of 75 inhabitants/km^2^, although this increases in urban areas (up to 1078 inhabitants/km^2^) (Tir 2024).

Rainfall records from 2023 and 2024, compared with the ten‐year monthly averages, reveal considerable variability between months and years within the period January to August. At the monthly scale, precipitation shows strong fluctuations. For example, January was extremely dry in 2023 (1 mm) but increased to 9 mm in 2024, although still below the long‐term mean of 16.7 mm. In contrast, May exhibited relatively high rainfall in both years (13 mm in 2023 and 21 mm in 2024), approaching the long‐term mean of 22.0 mm. The most striking contrast occurred in June, where 2023 was unusually wet (22 mm, more than double the mean of 10.2 mm), whereas 2024 was completely dry (0 mm). July and August were consistently very dry, with negligible values (≤ 4 mm), much lower than the respective averages of 3.9 mm and 7.5 mm. At the interannual scale, 2023 was characterized by generally low rainfall during the early months, followed by an exceptional peak in June, while 2024 displayed a more balanced distribution, with significant precipitation in January, February, May, and particularly June contrasting with its total absence. This indicates a high degree of irregularity, where one year concentrates rainfall in a single exceptional month and another year distributes rainfall more evenly across several months. Data taken from TuTiempo.net (https://fr.tutiempo.net/m‐sila.html).

### 2.2 Study Species

Only three previous studies were conducted on the breeding ecology of Barn Swallows in Algeria; none of them was in arid regions. The breeding season typically begins in the second half of April and extends until late July, although the exact timing may vary among years. Previous studies and our field observations indicate that while the onset of breeding is relatively consistent from year to year, interannual fluctuations in temperature and rainfall can shift laying dates by one to two weeks (Haddad 2012; Fenghour 2018).

### 2.3 Local Weather Data

Daily weather data (minimum, maximum and mean temperature, precipitation, and wind speed) were obtained from the nearest weather station to the study area, M'sila station (station number 604670), via the website TuTiempo.net (https://fr.tutiempo.net/m‐sila.html). The station is located ~5 km away from the central cluster of our subsites. Although our subsites span up to ~100 km, we used M'sila as the reference. For each nest, we identified the specific dates corresponding to the main breeding stages (i.e., egg‐laying period, incubation duration, nestling duration, and overall breeding periods). We defined the “egg‐laying period” as the 10 days preceding the laying of the first egg, based on known physiological requirements in Swallows (Saino et al. 2004). For each of these periods, we extracted daily weather data (temperature, precipitation, and wind speed) and calculated mean values for the different breeding stages of each nest. Given that in this area, water courses generally are ephemeral, we focused on short‐term effects of precipitation as it is likely that local organisms respond rapidly to changing weather conditions in arid environments (Maute et al. 2019).

### 2.4 Data Collection

Nest monitoring in our study started well before the anticipated onset of breeding activity, ensuring that first clutches were recorded. Nests were located through systematic searches across villages and farmland before the beginning of the breeding period. The species frequently reused nests from previous years. All nests were located on man‐made structures. Once located, each nest was monitored through at least two systematic visits per week (every 3–4 days) until either the nestlings fledged or the breeding attempt failed. We visited the nests more often near key dates (egg‐laying and hatching). We estimated the age of the nestlings if there were instances where we found all eggs hatched (e.g., looking at the degree of wetness of body feathers and the redness of the skin). While these were conservative estimates, this monitoring allowed us to record key reproductive parameters. The laying date of the first egg was determined by direct observation or estimated by back dating assuming Barn Swallows laid one egg per day. The clutch size was defined as the total number of eggs laid in a nest, recorded once no new eggs were added for at least two consecutive visits. Hatching success was defined as the number of eggs that successfully hatched divided by clutch size. The incubation duration was calculated as the number of days between the laying of the last egg and the hatching of the first chick. Failed nests were excluded from the calculation of incubation duration. The duration of the nestling stage was defined as the number of days between hatching and fledging, based on regular nest checks (Saino et al. 2004). The number of fledglings corresponded to the number of chicks that left the nest, determined by the absence of nestlings from a previously occupied nest near the expected fledging age, which we used to estimate breeding success (number of fledglings divided by clutch size). All nests included in the study were discovered before the laying of the first egg, allowing for complete monitoring of the breeding attempt.

### 2.5 Statistical Analysis

To assess the effects of weather conditions, date, and habitat on the breeding performance of Barn Swallows nesting in arid environments of Algeria, we fit generalized linear mixed models using the Template Model Builder (Kristensen et al. 2016). We ran six models sequentially using the studied breeding parameters as dependent variables (incubation duration, nestling stage duration, clutch size, hatching success, breeding success, and renesting probability). We first visually inspected the data to assess data error distribution and assessed the correlations between different weather variables. We found that min, max, and mean temperature were highly correlated across all breeding stages (> 0.70), so we only used max temperatures in our models. The model for clutch size included max temperature (°C), average winds (km/h), and total precipitation (mm) during egg laying, as well as date (n days since April 1st), habitat type (rural, i.e., outside villages, or urban, within villages), and brood number (1 or 2) as explanatory variables. We also included year (2023 or 2024) and site (14 subsites) as random factors. We used the 14 subsites instead of the three main locations because these 14 subsites were relatively homogenous breeding colonies. The model for incubation duration included the same variables, although weather variables were computed across the incubation stage. Failed nests were excluded from incubation duration analyses. The model for hatching success included the same variables as in the previous model in addition to incubation duration. The model for the duration of the nestling stage used weather variables computed during this stage. In this model, wind was excluded due to convergence issues. The model for breeding success was as the previous one but included both incubation and nestling stage duration and used weather variables computed across the entire breeding period. The model for renesting probability included nestling stage duration and weather variables computed during the entire nesting period. This model also included laying date, but this variable correlated strongly with temperature, so it was finally excluded.

We centered and scaled all numeric variables by subtracting the mean and dividing the obtained values by the standard deviation. We employed a Gaussian distribution in all models but renesting probability (0 or 1), for which we used a binomial error type. To avoid over‐parameterization, we attempted to implement multi‐model inference and model averaging, but this approach did not perform well. We thus used Akaike Information Criteria (AIC) scores to implement a backward model reduction approach and obtain minimum adequate models until removing variables did not entail reductions in AIC scores. We used standard diagnostic plots including Kolmogorov–Smirnov, overdispersion, and outlier tests to assess model performance. All models performed well. We used variation inflation factors lower than three to determine if there were collinearity issues (Zuur et al. 2010). In some instances, temperature and date were highly correlated, so we ran two models using each of these two variables and kept the best‐performing model based on its AIC scores.

All analyses were conducted in R v4.4.2 (R Core Team 2024). Data manipulation was performed with dplyr (Wickham et al. 2023). Generalized linear mixed models were fitted using glmmTMB (Brooks et al. 2017), and model diagnostics were implemented with DHARMa (Hartig 2023) and performance (Lüdecke et al. 2023).

## 3 Results

### 3.1 Weather Effects on Incubation and Nestling Period Durations

**FIGURE 3:**
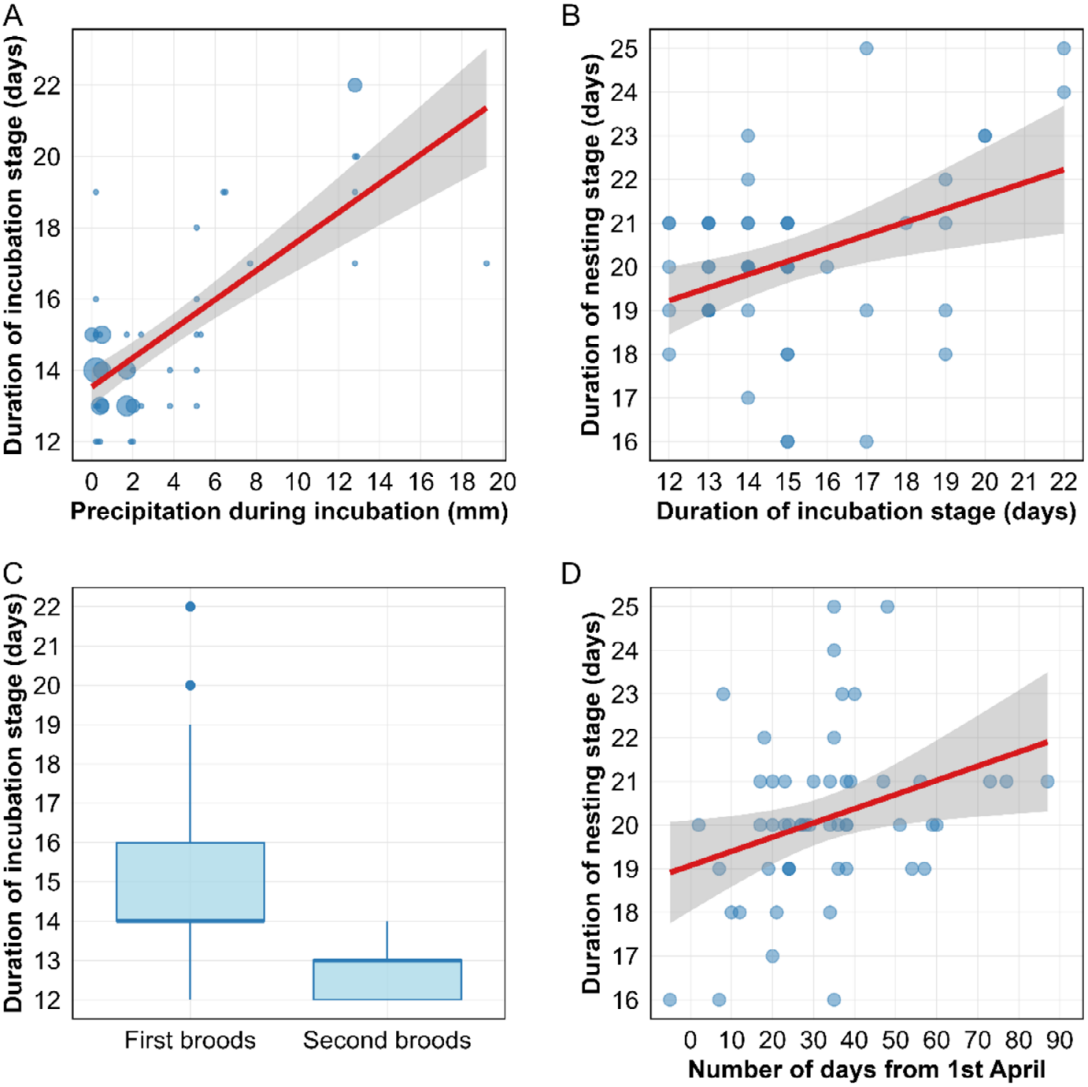
Relationships between weather variables and incubation and nestling period durations in Barn Swallows *Hirundo rustica* breeding in northern Argelia. (A) Linear relationship between precipitation during incubation and the duration of the incubation stage. Bubble size represents observation counts (1 to 6 observations according to increasing sizes). (B) Association between the duration of the incubation and nestling stages. (C) Comparison of incubation duration between first and second broods (boxplot shows median ± IQR). (D) Effect of breeding initiation date (n days since April 1st) on nestling period duration. Shaded areas in panels A, B, and D represent 95% confidence intervals and red lines are the regression lines.

The duration of the incubation stage correlated positively with precipitation recorded during the incubation period (Table 1, Figure 3A). No significant effects of temperature or wind on incubation duration were detected (Table A1). The duration of the nestling period was not affected by temperature, rainfall, or wind (Table A2). However, nestling period duration correlated positively with incubation duration (Table 1, Figure 3B). Incubation duration decreased in second broods (Figure 3C), but nestling period duration correlated positively with laying dates (Table 1, Figure 3D).

**TABLE 1:** Generalized linear mixed models using breeding parameters of Barn Swallows *Hirundo rustica* .

Predictor	Estimate	SE	*z* value	*p*	Random	Var.	SD
Incubation stage duration (AIC = 126.6)							
Intercept	< 0.001	0.1	0.09	0.92	Year	< 0.001	< 0.001
Precipitation	0.67	0.1	7.97	< 0.001	Site	< 0.001	0.001
Brood	−0.23	0.1	−2.84	< 0.001			
Nestling stage duration (AIC = 139.0)							
Intercept	0.01	0.1	0.14	0.88	Year	< 0.001	< 0.001
Incubation/duration	0.40	0.1	3.53	< 0.001	Site	< 0.001	< 0.001
Laying date	0.36	0.1	2.75	< 0.001			
Clutch size (AIC = 184.7)							
Intercept	< 0.001	0.1	< 0.001	1.00	Year	< 0.001	< 0.001
Laying date	−0.33	0.1	−2.84	< 0.001	Site	< 0.001	< 0.001
Hatching success (AIC = 111.0)							
Intercept	0.22	0.1	3.17	< 0.001	Year	< 0.001	< 0.001
Precipitation	0.14	0.1	1.94	0.05	Site	< 0.001	0.002
Brood	0.12	0.1	1.80	0.07			
Breeding success (AIC = 86.1)							
Intercept	0.38	0.1	5.46	< 0.001	Year	< 0.001	< 0.001
Precipitation	0.41	0.1	3.65	< 0.001	Site	< 0.001	0.002
Laying date	−0.27	0.1	−3.59	< 0.001			
Incubation/duration	−0.18	0.10	−1.68	0.09			
Renesting probability (AIC = 36.5)							
Intercept	−3.38	1.2	−2.77	< 0.001	Year	< 0.001	0.003
Precipitation	−2.33	1.6	−1.48	0.13	Site	< 0.001	0.100
Max Temp	−2.14	1	−2.18	0.02			

*Note:* The dependent variable is indicated for each model. We implemented model reduction by Akaike Information Criteria (AIC), removing independent variables until ΔAIC ≤ 2. We provide AIC score for each reduced model. Full models can be seen in Tables A1, A2, A3, A4, A5, A6. Year and subsite were included as random factors, and the variance explained by each random factor is provided for each model. Laying date was measured as n days since April 1st.

### 3.2 Effects of Incubation Duration and Weather on Reproductive Success

**FIGURE 4:**
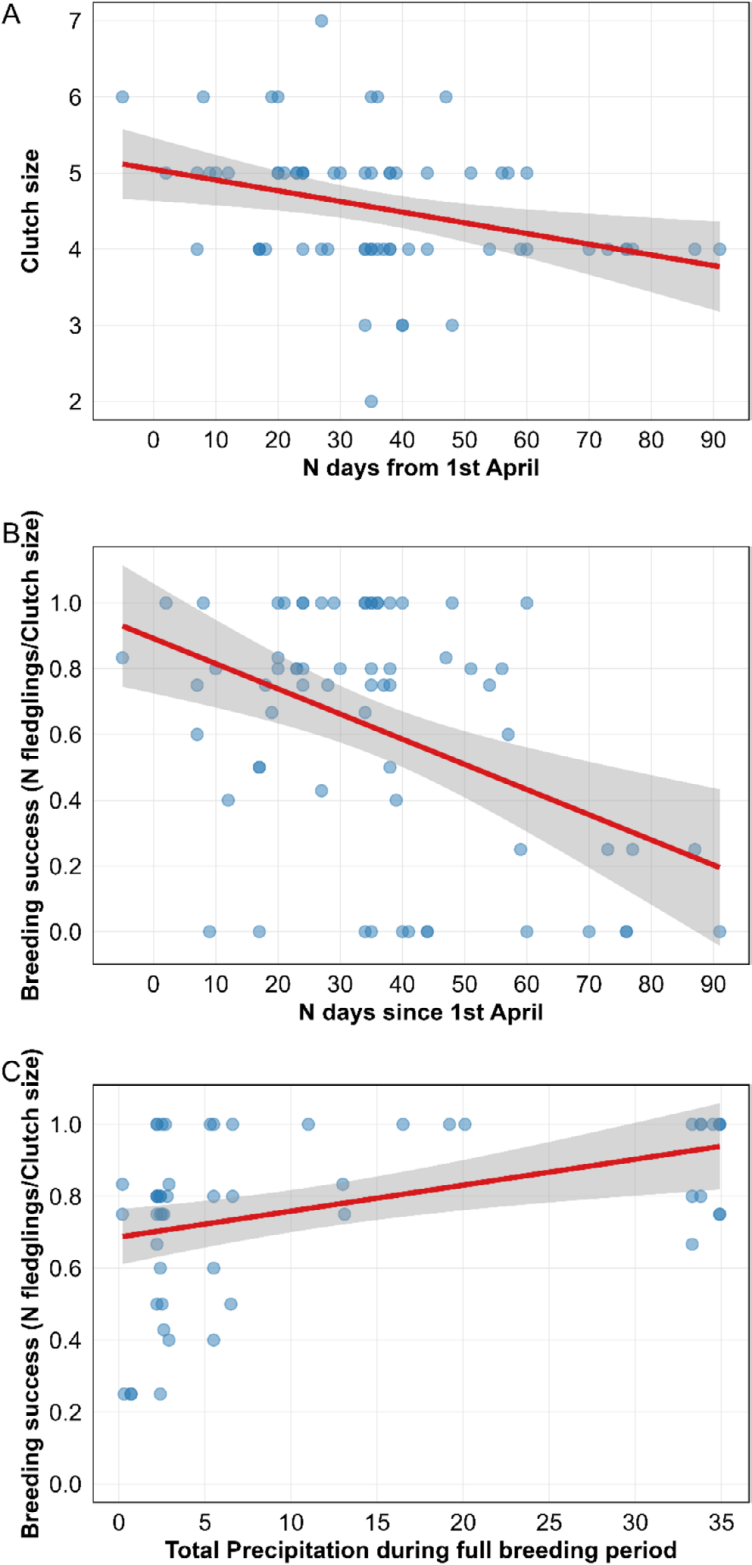
Relationships between seasonal timing, precipitation, and reproductive performance in Barn Swallows ( *Hirundo rustica* ) breeding in northern Algeria. (A) Linear relationship between breeding initiation date (n days since April 1st) and clutch size. (B) Association between breeding initiation date and breeding success (fledglings/clutch size). (C) Effect of total precipitation during the breeding period on breeding success. Shaded areas in all panels represent 95% confidence intervals and red lines are the regression lines.

Clutch size correlated negatively with laying date (Table 1, Figure 4A). Other climatic variables and habitat type showed no significant effects on clutch size (Table A3). Similarly, breeding success correlated negatively with laying dates (Table 1, Figure 4B).

Hatching success showed two non‐significant tendencies: to be higher in second than in first broods and to correlate positively with precipitation recorded during the incubation period (Table 1). Hatching success was not affected by temperature or wind (Table A4).

Breeding success correlated positively with total precipitation during the breeding period (Table 1, Figure 4C). In contrast, breeding success correlated negatively with both laying dates and incubation duration (Table 1). No effects of habitat type, temperature, or wind on breeding success were detected (Table A5).

### 3.3 Temperature Effects on Renesting Probability

Renesting probability correlated negatively with maximum temperature during the post‐breeding stage (Table 1). No effects of rainfall or wind on renesting probability were detected (Table A6).

## 4 Discussion

Our results show that local weather conditions significantly influence the reproductive biology of Barn Swallows in arid environments. Reproductive success showed a positive relationship with rainfall during the breeding period (Lloyd 1999; Skagen and Adams 2012) and declined progressively throughout the season and with extended incubation duration. We also observed that incubation duration increased with higher rainfall, while nestling period duration increased both with longer incubation and seasonal progression, suggesting potential synergies with other environmental factors in the effects of rainfall on breeding performance. Additionally, we identified a negative relationship between temperatures and renesting probability, suggesting that high temperatures limit Barn Swallows' reproductive efforts (Van de Ven et al. 2020). Therefore, our results suggest that local weather variables strongly influence reproductive strategies and the breeding performance of Barn Swallows in arid environments.

### 4.1 Precipitation Enhances Reproductive Success

Reproductive success increased with rainfall, indicating that even moderate increases in precipitation can alleviate food limitations in arid environments. Rainfall boosts insect availability in the mid‐term, which improves nestling growth and survival (França et al. 2020). This positive relationship confirms the importance of rain for reproductive success in insectivorous birds breeding in arid regions (Lloyd 1999). Our findings also show that rainfall was associated with extended incubation duration. Increased precipitation during the incubation period may reduce insect activity in the short term (Nooker et al. 2005; Coe et al. 2015), limiting food availability and leading to reduced incubation efforts. This suggests that precipitation has mixed effects on breeding, disrupting incubation behavior in the short term but increasing food abundance in the long term. Rain variability can also affect the ecology of insects. Heavy rainfall in a single month, such as June 2023, may create temporary breeding sites that allow short‐term population increases, but these are quickly lost in the following dry months. In contrast, when rainfall is spread across multiple months, as in early 2024, it leads to longer breeding seasons for insectivorous birds and their prey.

Intermittent incubation due to, e.g., precipitation or relatively high temperatures could result in slow embryonic development, thereby extending the incubation period duration. Similar relationships have been reported in other passerines such as the Eastern Kingbird (Gillette et al. 2021). Nestling duration was also positively associated with incubation duration, linking pre‐ and post‐hatching stages. Extended incubation may result in hatchlings with lower initial condition, requiring more time to reach fledging maturity (Ardia et al. 2010; Wuczyński 2012). Conversely, we found a negative relationship between incubation duration and reproductive success. Prolonged incubation may reflect suboptimal conditions such as poor weather or low food availability (e.g., during very rainy days), both of which can impair embryonic development and increase mortality risk (Diez‐Méndez et al. 2020; MacDonald et al. 2013). Long incubation periods may also affect parental energy reserves, reducing their ability to maintain optimal incubation temperatures or provide post‐hatching care.

### 4.2 Excessive Heat Limits Reproductive Efforts

Our results also indicate a negative effect of maximum temperature on renesting probability. High temperatures may increase adult thermal stress and energetic demands, reducing the likelihood of initiating a second clutch. In contrast to more humid subtropical regions where Barn Swallow have two and three broods (Pagani‐Núñez et al. 2016; Tian et al. 2022), these constraints likely are especially relevant in arid zones, where late‐season heat considerably reduces food availability and increases physiological stress (Du Plessis et al. 2012; Wingfield et al. 2017; Van de Ven et al. 2020). In arid zones, evapotranspiration is the main mechanism for heat dissipation of small passerines, which may compromise survival if humidity is very low and the costs of water loss become too high (Song and Beissinger 2020; McKechnie et al. 2021). In extreme events, heat can disrupt incubation and lead to brood failure even in highly specialized desert birds (McCowan and Griffith 2021). Reduced renesting probability under high temperatures likely reflects a strategic shift toward self‐maintenance over reproduction under challenging conditions.

Additionally, we found no significant effect of wind speed on breeding parameters of the Barn Swallow in this arid environment. This result contrasts with the findings of Møller (2013), who reported that increasing wind speed reduces insect abundance and consequently decreases reproductive success in Barn Swallows. Møller (2013) described a non‐linear relationship, where insect availability—and consequently foraging success—is significantly higher at low wind speeds (0–2 m/s) and declines sharply as speeds approach 5–6 m/s. In our study, the recorded wind speeds (5.53–7.03 m/s) fell within this higher, potentially inhibitory range. It is plausible that within this range, the aerial insect density is already reduced to a consistently low level that uniformly constrains foraging success. Also, in line with previous studies (Zhao et al. 2022; Chen et al. 2025), we found no significant effects of habitat type (urban vs. rural) on the breeding parameters studied, probably due to the predominantly rural nature of the study area.

### 4.3 Seasonal Decline in Reproductive Success

We also observed a negative relationship between laying date and clutch size. Early breeders produced larger clutches, consistent with a seasonal decline in reproductive investment. This may be related to older individuals arriving earlier to the breeding grounds than younger individuals (Balbontin et al. 2007). This trend is common in arid zones where environmental conditions deteriorate as the season advances. Early in the season, favorable temperatures and higher food availability (i.e., a peak in aerial insects' abundance, see, e.g., Tigar and Osborne 1997) may support larger broods, while later conditions may constrain reproductive effort (Harriman et al. 2017; Liu et al. 2024). Similarly, the increase in both incubation and nestling durations with seasonal progression may reflect declining food availability later in the breeding season, as insect abundance decreases with rising temperatures and increasing aridity (Teglhøj 2017; Gorosito et al. 2022; Murphy et al. 2022), suggesting that seasonal environmental constraints limit reproductive efforts. Breeding success also declined across the season, likely due to the combined effects of thermal stress and reduced provisioning efficiency (Ambrosini et al. 2006; Włodarczyk and Minias 2020). These results support the idea that breeding earlier confers a fitness advantage under arid conditions.

### 4.4 Potential Impacts of Climatic Change

Recent projections indicate that Algeria has warmed substantially in recent decades, and many regions already face declining rainfall, increased drought frequency, and greater aridity (Derradji 2023). These climatic changes are expected to intensify under future climate change scenarios. Very high temperatures can reduce embryonic viability, slow nestling growth, and increase mortality (Conradie et al. 2019). Moreover, warming and drought are likely to reduce insect abundance, leading to poorer body condition, smaller offspring, and potentially breeding failure. In arid zones, reproductive success may also decrease during hot years if food or water becomes a limiting factor late in the breeding season (Maute et al. 2019). Droughts can occasionally occur in early spring, including April, coinciding with the onset of breeding in Barn Swallows, which may further constrain reproductive performance in these environments.

### 4.5 Study Limitations and Future Perspectives

While this study provides valuable insights into how local climatic conditions affect the reproductive success of Swallows in arid environments, several limitations must be acknowledged. First, food availability was not directly measured. Monitoring insect abundance throughout the breeding season would have provided a clearer understanding of the link between precipitation and reproductive outcomes (Tigar and Osborne 1997). Second, the age and breeding experience of adults, factors known to influence the success of second broods and the likelihood of renesting, were not assessed (Martin 1995). Future research should incorporate detailed evaluations of the physiological condition and age structure of breeding individuals. Third, our findings are based on data from only two breeding seasons, which may not fully capture inter‐annual variability. Long‐term, multi‐year studies are essential to better evaluate the potential effects of ongoing climate change on reproductive trends in arid regions.

## 5 Conclusion

This study highlights the major influence of local climate conditions on the reproduction of Barn Swallows in arid zones. We demonstrated that precipitation enhances reproductive success by increasing food availability, while excessive heat limits reproductive effort and reduces the renesting probability (Van de Ven et al. 2020). Furthermore, we observed a seasonal decline in reproductive success, with later clutches being associated with longer incubation duration and lower hatching rates (Murphy et al. 2022). These results underscore the critical role of local weather factors in shaping the population dynamics of Barn Swallows, particularly in arid environments where environmental conditions are especially challenging. In the context of climate change, rising temperatures and altered precipitation patterns may reduce the reproductive success of this species, potentially threatening its long‐term viability (Ma et al. 2023). It is therefore essential to monitor the evolution of Swallows' populations and assess the adaptive strategies they may adopt in response to environmental changes. Additional studies incorporating physiological and behavioral data would help deepen our understanding of the mechanisms underlying these reproductive responses. Our research contributes to a better understanding of avian reproductive ecology in arid environments and could inform conservation actions tailored to the impacts of climate change on migratory birds.

## Author Contributions

**Fatima Gherbaoui:** investigation (lead), writing – original draft (equal). **Taqiyeddine Bensouilah:** conceptualization (lead), data curation (lead), formal analysis (equal), writing – original draft (lead), writing – review and editing (equal). **Emilio Pagani‐Núñez:** conceptualization (equal), data curation (equal), formal analysis (equal), writing – original draft (equal), writing – review and editing (equal). **Moussa Houhamdi:** investigation (lead), writing – review and editing (equal).

## Conflicts of Interest

The authors declare no conflicts of interest.

## Data Availability

Data are provided as a supplement and will be made publicly available upon acceptance.

## Appendix 1

**TABLE A1:** Full model of incubation duration.

Predictor	Estimate	SE	*z* value	*p*
Intercept	0.07	0.11	0.66	0.50
Wind	−0.03	0.08	−0.36	0.71
Precipitation	0.70	0.09	7.87	< 0.001
Temperature Max	0.13	0.11	1.21	0.22
Habitat type	−0.13	0.16	−0.80	0.42
Brood	−0.30	0.10	−2.93	0.00

**TABLE A2:** Full model of the duration of nestling stage.

Predictor	Estimate	SE	*z* value	*p*
Intercept	−0.11	0.17	−0.64	0.52
Precipitation	−0.00	0.18	−0.01	0.98
Incubation duration	0.39	0.18	2.17	0.03
N days since April 1st	0.39	0.16	2.36	0.01
Number of hatchlings	−0.04	0.17	−0.27	0.78
Brood	−0.09	0.17	−0.56	0.57
Habitat type	0.26	0.24	1.10	0.26

**TABLE A3:** Full model of clutch size.

Predictor	Estimate	SE	*z* value	*p*
Intercept	−0.04	0.16	−0.24	0.81
Temperature Max	−0.13	0.24	−0.57	0.57
Wind	0.02	0.15	0.14	0.89
Precipitation	0.02	0.13	0.15	0.88
N days since April 1st	−0.31	0.24	−1.27	0.21
Habitat type	0.08	0.24	0.33	0.74
Brood	0.13	0.16	0.84	0.40

**TABLE A4:** Full model of breeding success.

Predictor	Estimate	SE	*z* value	*p*
Intercept	0.39	0.10	3.76	< 0.001
Wind	−0.05	0.07	−0.68	0.49
Precipitation	0.40	0.11	3.56	< 0.001
N days since April 1st	−0.28	0.10	−2.68	< 0.001
Nestling duration	0.01	0.08	0.12	0.90
Incubation duration	−0.18	0.11	−1.59	0.11
Habitat type	−0.02	0.14	−0.18	0.85
Brood	0.01	0.09	0.18	0.85

**TABLE A5:** Full model of hatching success.

Predictor	Estimate	SE	*z* value	*p*
Intercept	0.22	0.10	2.19	0.02
Temperature Max	0.02	0.10	0.26	0.78
Wind	0.04	0.07	0.56	0.57
Precipitation	0.16	0.11	1.44	0.14
Clutch size	0.09	0.08	1.02	0.30
Incubation duration	−0.00	0.11	−0.02	0.97
Habitat type	−0.01	0.14	−0.08	0.93
Brood	0.12	0.09	1.24	0.21

**TABLE A6:** Full model of renesting probability.

Predictor	Estimate	SE	*z* value	*p*
Intercept	−3.62	1.56	−2.30	0.02
Wind	0.65	0.57	1.13	0.25
Precipitation	−2.63	2.05	−1.28	0.19
Temperature Max	−1.73	1.01	−1.71	0.08
Nestling duration	0.28	0.81	0.34	0.73
Habitat type	0.59	1.12	0.52	0.59
